# Knowledge, Attitudes, and Practices of Parents in the Use of Antibiotics: A Case Study in a Mexican Indigenous Community

**DOI:** 10.3390/healthcare12030294

**Published:** 2024-01-24

**Authors:** Ana María González-Villoria, Alma Delia García Quiroz, Edgar Ulises Osorio Guzmán, José Carlos Suarez-Herrera, Roberto Ariel Abeldaño Zuñiga

**Affiliations:** 1 Postgraduate Department, Universidad de la Sierra Sur, Oaxaca 70800, Mexico; ana.gonzalez@unsis.edu.mx (A.M.G.-V.);; 2 Benemérito Instituto Normal del Estado General Juan Crisóstomo Bonilla, Puebla 72140, Mexico; osorio.guzman.eu@bine.mx; 3 UNITWIN/UNESCO IPD-SILOS, University of Las Palmas de Gran Canaria, 35001 Las Palmas de Gran Canaria, Spain; joseko70@hotmail.com; 4 Centre for Social Data Science, Faculty of Social Sciences, University of Helsinki, 00100 Helsinki, Finland

**Keywords:** antibiotic resistance, traditional medicine, social factors, biological–social analysis, alternative treatments, vulnerability

## Abstract

The rise and spread of antibiotic-resistant bacteria have become a global health problem. At the community level, bacterial resistance has been linked to antibiotic misuse practices. These practices are related to social factors such as education level, poverty, ethnicity, and use of traditional medicine. Through a survey, this study aims to analyse the knowledge, attitudes, and practices (KAP) of antimicrobial use, in an indigenous community in the south of Oaxaca, Mexico. It was observed that the population had a low socioeconomic profile, poor access to healthcare services, low academic level, little knowledge of antibiotics, the use of traditional medicine, and proper attitudes and practices regarding antibiotics use. Therefore, social factors are related to bacterial resistance only if they make the population prone to the use of antimicrobials. Lack of medical access and cultural factors drives this population to use ancestral alternatives such traditional medicine to treat conditions that in other contexts could be treated with antibiotics. This is an example of how the population can reduce the consumption of antimicrobials in infections if they have a reliable alternative that improves their symptoms.

## 1. Background

Antimicrobial resistance, which has increased alarmingly in recent decades, is a phenomenon that places the health of the world’s population at risk by weakening the treatment of infectious diseases and increasing mortality and healthcare costs [1].

Previous studies have related the rise of this problem to community practices such as irrational consumption, interruption of medical treatments, self-medication, socioeconomic factors, and lack of access to health services in developing countries, particularly concerning pathogenic bacteria in the respiratory tract [2,3]. It has been reported that in developing countries, social conditions and behaviours often result in the inappropriate use of antimicrobials, and the prevalence of resistant bacteria is very common [3]. An example of misuse of antimicrobials is the treatment of upper respiratory tract infections, where antibiotics are indiscriminately prescribed to address these conditions, but are unnecessary in a third of these cases, as 50% are of viral origin [4,5]. The paediatric population is the most susceptible to receiving antimicrobials due to the following two situations reported by paediatricians: (1) they worsen rapidly, especially in winter and cold weather, and (2) the parents ask for the medication [6,7]. It is known that the way parents react to a disease can determine its outcome, as practices related to the misuse of antibiotics can contribute to the development of resistant bacteria [7].

But what about populations in conditions of vulnerability, without access to health services, who understand that instead of indiscriminately consuming antibiotics, they use traditional medicine? This medicine is also called home remedies, which are widely used to treat diseases, and are considered the first level of health care [8]. What are the knowledge, attitudes, and practices (KAP) in these conditions? To answer this question, a study was conducted in an indigenous community in Mexico that meets the socioeconomic and health services characteristics commonly related to bacterial resistance. A KAP questionnaire was administered to elucidate whether poverty conditions and community practices were related to the presence of resistant bacteria.

## 2. Methods

### 2.1. Description of Population

The Mexican state of Oaxaca has one of the highest numbers of indigenous ethnic groups facing challenges in accessing health services, but also possesses extensive knowledge in the use of traditional medicine. This state is a favourable territory to determine whether the behaviour in situations of healthcare inaccessibility is similar to that reported in other low-income communities in terms of the pattern of antimicrobial use.

The population under analysis belongs to communities in the state of Oaxaca, Mexico, situated in mountainous areas between 2300 and 2662 m above sea level, so they experience predominantly cold weather. Overall, 87% lived with a high degree of social marginalisation, 21% did not have access to health care services, 85.6% lacked social security, and approximately 30% had a low educational level [9], placing them as a population with high vulnerability to bacterial resistance [10]. The communities had two medical clinics. One is the Mexican Social Security Institute Medical Unit (IMSS-BIENESTAR), and the other belongs to the Ministry of Health (SESA), with two family physicians per unit (0.7 physicians per inhabitant) [11,12,13] Regarding income, 91.7% were below the poverty line, and 67.9% were below the minimum poverty level.

**Figure 1:**
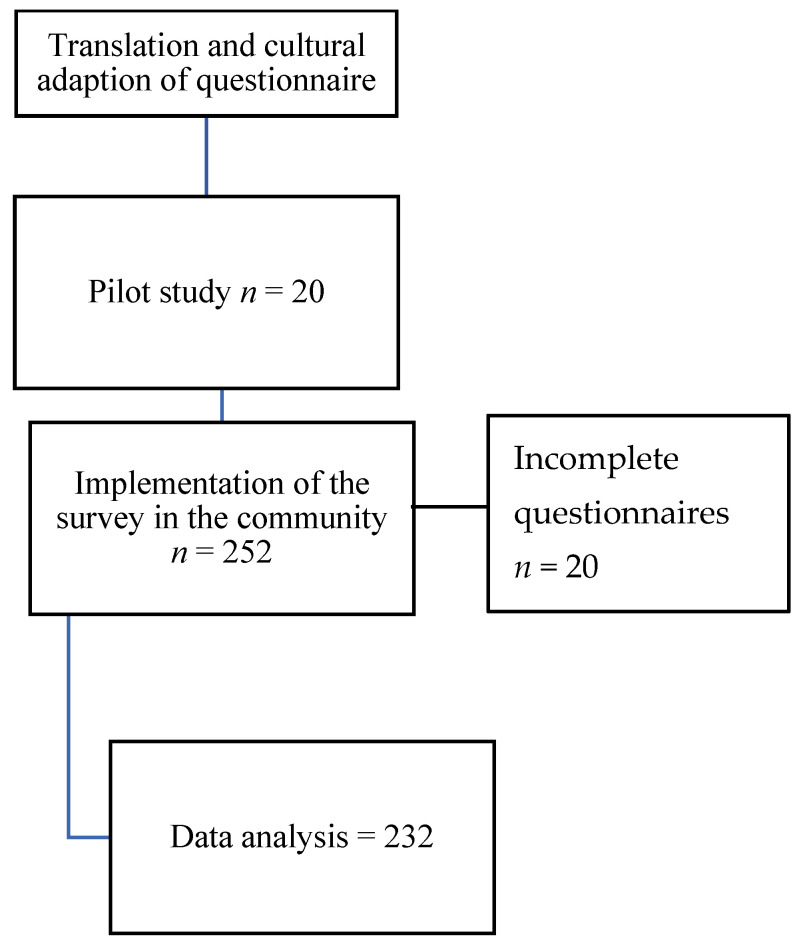
Flowchart of the study.

A cross-sectional study was conducted in three primary schools and four pre-schools in January, May, June, and November 2019. The inclusion criteria were parents who agreed to participate. The exclusion criteria were those whose parents did not complete the questionnaire entirely. Finally, a final sample (*n*) of 232 KAP questionnaires was obtained (Figure 1).

All participants signed an informed consent prior to the study, and data were protected in the study by assigning consecutive numbers to the names. The fieldwork consisted of a survey, including a KAP questionnaire and open-ended questions about the use of traditional medicine in infections, administered to the parents. All participants included in the study spoke Spanish.

#### 2.1.1. Knowledge, Attitudes, and Practices Questionnaire

First, the authors conducted a translation from English into Spanish, and a back-translation from Spanish into English by a language specialist, along with a cultural adaptation of the questionnaire developed by Ding et al. [14] (see Supplementary Materials).

Initially, a pilot study was conducted with 20 individuals, where the response options for items 17–20 in the knowledge section and items 21–28 in the attitudes section were modified. Closed-ended questions with multiple-choice answers were considered most suitable for ease of analysis, and the following response options were chosen:

- Totally disagree;
- Disagree;
- Undecided;
- Agree;
- Totally agree.

The question “What illnesses did you have?” originally had an open-ended nature, so the following response options were added: respiratory illnesses, gastrointestinal illnesses, and others. Additionally, the items “How many bedrooms does the house have for sleeping?” and “What type of home remedy do you use to treat the flu?” were added.

The questionnaire includes four sections. The first section includes sociodemographic characteristics. The following three sections assess the level of knowledge (19 items) regarding antibiotic identification and knowledge about treatment with medical prescriptions. One point was assigned for agreement with positive statements, and zero points were given for disagreement. The scores were categorised as high (12–18 points), medium (6–11 points), and low (0–5 points). The attitudes section consists of 15 items. Attitudes were classified as positive if 4–7 appropriate responses were given and negative if 0–3. The practices section includes 12 items, with practices categorised as positive if there are 7–12 appropriate responses and negative if 0–6. The Cronbach’s alpha for the current study was 0.918 for the items included in the knowledge section, 0.774 for the attitudes section, and 0.635 for the practices section.

The operational definitions used in the questionnaire were as follows: “Knowledge” can be conceptualized as the level of awareness and understanding individuals have about a specific topic. “Attitudes” are understood as how people feel about a particular topic and the preconceived ideas or beliefs they may have about the topic. Finally, “Practices” are defined in this study as how people apply their knowledge and attitudes about a specific subject through their actions [15].

We considered adherence as defined by the World Health Organization, Adherence is “the extent to which a person’s behaviour-taking medication, following a diet and/or executing lifestyle changes, corresponds with agreed recommendations from a healthcare provider” [16], while expectation “can be thought of as a prediction about the consequences of certain health-related phenomena (behaviours and conditions, both internal and external), on the psychological condition of the body” [17].

The level of education refers to the average number of years attained in formal education by a group. In Mexico, the population over 15 years old has an average of 9.7 years of formal education.

#### 2.1.2. Statistical Analysis of the Data

The categorical data were compared using the χ^2^ test with 95% confidence intervals. *p* values 0.05 were considered as statistically significant. STATA software version 22 was used to analyse the data.

## 3. Results

### 3.1. Sociodemographic Characteristics

The income level of the population was within the first and second quartile deciles according to the National Institute of Statistics and Geography (INEGI), at 6820 and 12,350 Mexican pesos, respectively [18]. Of these households, 51.4% lived in moderate to critical overcrowding conditions. The statistics indicated that 64.7% of the caregivers were aged 35 or younger, 89.7% of those who completed the questionnaire were women, and 78% of the participants had a moderate level of education, lower than the national average of 9.7 years of formal education per person [19].

The children had an average age of 6.23 years, with 50.4% being male and 49.6% female. Parents reported that 61.0% of their children had a respiratory tract illness at least once in the last six months, 13.0% had a gastrointestinal illness, and 11% had other illnesses. They perceived that their child’s health was good.

### 3.2. Knowledge

The information collected in this section is mainly related to the recognition and method of obtaining antimicrobial agents. Knowledge about antibiotics was determined by asking the parents if they had heard the name and if they knew which were antibiotics from a list. Only 2.32% of participants correctly identified all the antibiotics. The most recognized were penicillin (65.9%), amoxicillin (57.3%), and metronidazole (34.1%). The knowledge shown by the participants was linked to age range, with adults under 35 years mostly identifying cephalosporins (cephalexin and ceftriaxone), while those older than 35 recognized β-lactams (ampicillin and penicillin).

More than half of the participants (58.6%) knew that a prescription is necessary to obtain an antibiotic, and 56.7% said that they followed the doctor’s instructions when an antibiotic was prescribed. Additionally, 57.8% agreed with the recommendation that using antibiotics inappropriately can create resistance. The primary means of information that caregivers had access to regarding the use of antibiotics were doctors, followed by the leaflets contained with the medications, and advice given by pharmacists.

### 3.3. Attitudes

The evaluation considered whether parents’ attitudes were in favour of or against the use of antibiotics in situations such as taking antibiotics to prevent flu, combining antibiotics for a better effect, stopping antibiotic treatment when symptoms improved, believing that the most expensive antibiotics are better, asking the doctor to prescribe an antibiotic for their child, and knowing that a prescription is necessary to acquire an antibiotic. It was found that 53.4% of the participants had a positive attitude. Additionally, 29.3% believed that it was necessary to use an antibiotic when the infant had a fever, 24.6% for an ear infection, and 23.7% for a sore throat. Among these, 52.8% of respondents stated that antibiotics should be used for more than two conditions, indicating high expectations for the use of antibiotics (Table 1).

### 3.4. Practices

#### 3.4.1. Use of Antibiotics in the Community

The questions focused on the main behaviours associated with resistance [10] (as previously described in the background section), such as self-medication, antibiotic storage, related to negative practices and adherence to treatment, and the correct use of antibiotics related to positive practices.

Overall, 57.1% of the participants did not keep antibiotics at home, while 31.2% saved them, and 11.7% were not sure if they had antibiotics. It was found that 66.5% of the participants have positive practices, and 33.47% have negative practices regarding the use of antibiotics.

When asked about the names of the last antibiotics used, they mentioned active ingredients like penicillin, amoxicillin, paracetamol, and erythromycin, but they also mentioned the trade names of antipyretics, anti-inflammatory drugs, or dewormers.

Among caregivers, 90.5% said they acquired antibiotics by medical prescription. Once they had been prescribed an antibiotic, 61.5% had high adherence to treatment, and 78.5% did not increase the dose to make it more effective (see Table 1).

#### 3.4.2. Use of Home Remedies

It is important to note that in this study, 70.3% of the participants report using some type of home remedy included in their own traditional medicine to treat respiratory tract infections. If the remedy does not improve the symptomatology, then they go to the doctor. Among those used orally were several teas. The teas used can contain a single herb such as mullein tea (*Verbascum thapsus*), honey with lemon, and a combination of several herbs or flowers. For example, “to cure the flu thyme tea, ginger, bougainvillea is used” (*Zingiber officinale*, *Bougainvillea*).

Among the topical remedies to treat the symptoms of upper respiratory tract infections are mentholated ointment applied on the chest, back, and nose, and mezcal baths (a local alcoholic beverage made from agave) are used for a runny nose; mezcal is also used in a bath with urine, and mezcal is applied on the soles of the feet and the soft spot (fontanel) of babies. Tomato (*Physalis philadelphica*), when heated, cures “by applying tomato onto arms and the throat. For ear pain, apply basil with coconut oil”.

### 3.5. Association Analysis

The sociodemographic characteristics and use of home remedies were compared with expectations, adherence to treatment, and social factors related to bacterial resistance, knowledge, attitudes, and practices.

The association of sociodemographic and other characteristics with expectations was not significant. Adherence was significantly associated with persons under 35 years (*p* = 0.002) and with a medical prescription for considering the use of the antibiotic (*p* = 0.002) (see Table 1).

Attitude was significantly associated with the age of the caregiver under 35 years old (*p* = 0.006) and good practices (*p* = 0.003). Regarding the use of home remedies, there is no significant association with any variable (see Table 2).

## 4. Discussion

The aim of this study was to analyse and determine whether there was a relationship between the KAP in an indigenous community whose first option was to use traditional medicine over antimicrobials.

Upper respiratory tract infections (URIs) are the principal diseases associated with the misuse of antibiotics, as they are prescribed unnecessarily due to their mostly viral origin. In low-income populations, some studies found associations between socioenvironmental risk factors and the development of acute and recurrent respiratory tract infections [20,21].

### Social Conditions Related to Bacterial Resistance Increase

In this study, half of the population lived in overcrowded conditions. Overall, 82.8% of those living in overcrowding have been ill at least once in the last six months, and 8.2% of them have been ill more than three times. This frequency is lower than that reported in other studies, which ranged from six to ten upper respiratory tract infections (URIs) per year [20,21,22]. Therefore, this population, which did not have a high number of diseases, did not use antibiotics more frequently than the rest of the population. The level of formal education in most of this population was lower than the national average (9.1 years). In this community, the population had a positive attitude towards antibiotic use, similarly to people with a higher level of education reported by other studies [23,24].

This situation shows that in this sample, social factors, including poverty, do not necessarily translate into a lack of protection against possible respiratory infections; on the contrary, poverty, lack of medical access, and cultural factors drive this population to use an ancestral alternative such as traditional medicine to treat conditions that, in other contexts, could be treated with antibiotics.

One of the crucial points of the proposals for the containment of bacterial resistance suggested by the WHO is to increase knowledge [25]. In this study, although a high percentage of the population recognized penicillin, amoxicillin, and metronidazole, only 2.32% of the population correctly identified most of the antibiotics included in the questionnaire. Therefore, the study confirms that this population has a low level of antimicrobial knowledge about antibiotics, as similarly shown in other studies. However, unlike Pavydė’s study, a low level of knowledge of antimicrobials was not associated with inappropriate behaviour [26].

In Mexico, since 2010, the sale of antibiotics with a medical prescription has been decreed in the official gazette of the federation. The aim was to reduce antibiotic consumption, and the emergence of clinics near pharmacies has been observed, along with a change in consumption patterns; however, this is notwithstanding the situation in rural areas.

In 2018, the mandatory implementation of the National Strategy for Action against Antimicrobial Resistance was declared. This strategy aims at establishing a program of evidence-based educational communication. For this purpose, in line with action, the level of knowledge of antimicrobial resistance should be estimated [27]. The interventions implemented in Mexico are directed at healthcare personnel [28], while for the general population, there are some informational pamphlets [29].

For example, in Greece, parents rarely request antibiotics [30]; however, in other contexts like Singapore, parents consider that antibiotics cure more quickly, and this is associated with the level of education [31]. On the other hand, in other Latin American communities, parents have little knowledge on the correct use of antibiotics, unlike the parents in this study [32].

Health beliefs lead this population to use ancestral treatments as a first choice that show no relationship with the increase in antibiotic resistance, generating a protective factor against antimicrobial resistance. Therefore, bacterial resistance should not be directly related to the lack of pharmacological knowledge of the population, but to more complex contextual factors, such as lack of access to healthcare services, poor medical prescription, lack of alternative treatments, health governance.

The positive attitude towards what the physician prescribes was not found to be related to socioeconomic conditions or knowledge about antibiotics, since most of the population did not agree to request antibiotics from physicians when it was unnecessary, demonstrating a positive attitude. This is similar to the results of a study conducted in Sweden, which showed that most of the respondents show an appropriate and restrictive attitude towards antibiotics [33]. The population has limited access to healthcare services as well as to the media, so they do not miss an antibiotic for their treatment, which has been used indiscriminately with the biomedical model, even as a prophylactic to prevent infections. An important behaviour conducive to bacterial resistance is self-medication and keeping antibiotics at home [34]. In this study, the lack of healthcare services did not lead to reported self-medication practices or storage of medicines at home. One-third effectively stated that they used some kind of home remedy to treat the flu, in the same way as reported in other regions of Mexico where healthcare services are lacking [35].

Since, in 80% of cases, respiratory tract infections are viral in nature, this makes the use of antibiotics for treatment unnecessary. This suggests that the use of antimicrobials only when necessary could be related to the low levels of antimicrobial resistance, as recommended by the WHO [36].

In areas with low accessibility to healthcare services, the practice of traditional medicine serves as an alternative to reduce the use of antibiotics [37]. As in the case of a viral infection, the use of traditional medicine contributes to the symptomatic care of respiratory conditions. This could result in the low presence of resistant bacteria compared to people who, for various reasons, make greater use of antibiotics, leading to bacteria with greater resistance. Therefore, the use of traditional medicine can be an example of an alternative treatment for the symptomatic control of viral respiratory diseases to reduce the unnecessary use of antimicrobials, mainly in viral infections, such as the use of aromatic plants in upper respiratory tract infection symptoms [38,39,40,41].

In our study, we recognize the importance of alternative models of healthcare provided by traditional healthcare institutions and healthcare professionals, not recognized by the biomedical model, where providers are as diverse as culture determines [42]. The use of traditional medicine is related to different causal phenomena, such as a lack of access to healthcare services and cultural customs [43]. The conditions of this study population have unintentionally led them to follow the recommendation of not using antibiotics indiscriminately.

Among the actions to manage antibiotic resistance is home care, avoiding the development of nosocomial infections that require excessive use of antibiotics. Doctors and nurses should adopt antimicrobial management strategies at home, utilizing digital tools or telemedicine, with new treatment opportunities and therapeutic choices [44,45,46].

Therefore, surveillance studies of resistant bacteria should be carried out locally with appropriate treatment guidelines in regions with health disparities and not be excessively influenced by reports from hospitals or different communities that stigmatize indigenous communities as having inappropriate behaviours regarding antibiotics and high levels of bacterial resistance. This misguided attitude could increase the utilization of high-spectrum antibiotics by healthcare personnel in empiric treatment, as low socioeconomic levels are usually equated with high levels of bacterial resistance. In fact, the use of traditional medicine could be a way to improve adequate behaviour with respect to the antibiotics used, especially topical remedies to treat symptomatology. Phyto-pharmacological studies show that medicinal plants that have been used for centuries have positive effects on the symptomatology used because these plants have been noted for their anti-inflammatory activity, antioxidants, antibacterial [47,48,49], or oregano (*Origanum vulgare*) tea to treat coughs, this plant have antimicrobial properties [50]. Other studies reported that this effect occurs through different mechanisms of action compared to those of antibiotics, with no specific targets [51].

Consequently, it seems to us that it is necessary to reconsider whether the behaviour of the population depends exclusively on variables such as the level of knowledge, attitudes, or practices. Should we analyse what kind of knowledge is required in the population? Do they need to recognize antibiotics or understand how to act when faced with an infection and how to use antibiotics correctly? This is important for targeting campaigns and not getting lost in ambiguities such as “increasing knowledge.” Restructure social indicators only if they make the community more prone to antibiotic use, which is the primary mechanism for generating and disseminating bacterial resistance. This finding has been demonstrated in various studies in public health [52,53]

A limitation to consider in the study is that the population is immersed in the PROSPERA social inclusion program where they must fulfil health responsibilities, that is, attend consultations and workshops to receive monetary support, so the responses could be influenced by belonging to this program [54]. Another limitation is related to the small size of the study sample, which, along with the particular characteristics of the local culture, makes the reported results not generalizable to the entire population but only locally impactful. The third limitation we can mention is related to the cross-sectional design of the study, which does not allow causality to be established. On the other hand, in this study, only the knowledge, attitudes, and practices reported by one of the parents of the children were obtained, not both; therefore, there could be differences between the knowledge, attitudes, or practices of both parents that could not be retrieved by this study.

## 5. Conclusions

In this study, the socioeconomic status, knowledge, attitudes, and practices regarding the use of antibiotics were investigated. The social conditions were directly analysed, obtaining results different from those previously reported. Although the population had a low socioeconomic profile, poor access to healthcare services, a low academic level, and little knowledge of antibiotics, it was not found that they misused them. Additionally, they displayed proper attitudes and practices regarding antibiotic use.

The study of the association between the level of education and knowledge of antibiotics and the practices used so far is too reductionist to determine that a given indigenous population with few years of formal education is a determining factor in the creation and dissemination of resistant bacteria. The socio-sanitary factors associated with bacterial resistance are an essential part of the study of the emergence and dissemination of resistant bacteria, as previously described. Consequently, these conditions must be directly related to the use of antimicrobials, regardless of whether they occur in rural, urban, hospital, or community settings.

This situation shows that among the priorities of Global Health, we have the need to rethink the Western conception of health and risk factors, which, although they provide a statistical association, are neither determinant nor sufficient to explain certain complex phenomena [10,55] This could be achieved by incorporating transdisciplinary studies, integrating social sciences with health sciences, generating a broader and more complex approach to population health.

## Figures and Tables

**Table 1:** Association of characteristics of the participants and expectations and adherence.

Sociodemographic Characteristics		Expectations%		*p*	Adherence%		*p*
		Low	High		Low	High	
Age of caregiver	≤35 years	52.9	47.1	0.663	30.9	69.1	0.002 *
	≥36 years	48.6	51.4		52.7	47.3	
Sex of caregiver	Male	46.2	53.8	0.615	45.5	54.5	0.459
	Female	53.5	46.5		37.4	62.6	
Educational level	Basic	56.1	43.9	0.309	40.3	59.7	0.308
	High school	46.7	53.3		32.8	67.2	
Income level	Decile I	54.2	45.8	0.785	38.6	61.4	0.436
	Decile II	58.3	41.7		50.0	50.0	
Relationship to child	Parent	54.5	45.5	0.070	38.5	61.5	0.722
	Other	16.7	83.3		33.3	66.7	
Number of children	1	53.5	46.5	0.811	34.3	65.7	0.116
	≥2	51.2	48.8		45.0	55.0	
Age of child in this study	3–5 years	57.1	42.9	0.532	33.3	66.7	0.321
	6–8 years	51.2	48.8		40.3	59.7	
Sex of child in this study	Male	50.8	49.2	0.660	38.7	61.3	0.864
	Female	54.7	45.3		37.6	62.4	
Overcrowding	Moderate	54.2	45.8	0.488	37.5	62.5	0.365
	Critical	44.4	55.6		29.1	70.9	
**Other characteristics**							
Use of home remedies	Yes	60.0	40.0	0.391	40.6	59.4	0.633
	No	51.0	49.0		37.2	62.8	
Factor when considering antibiotic use	Medical prescription	54.5	45.5	0.412	35.1	64.9	0.002 *
	Other	42.9	57.1		72.2	27.8	
Adherence	Low	33.8	66.2	0.412	-	-	-
	High	41.1	58.9		-	-	

* Statistically significant.

**Table 2:** Association of characteristics of the participants with knowledge, attitudes, and practices.

Sample Characteristics		Knowledge			*p*	Attitude		*p*	Practices		*p*
		Low	Medium	High		Negative	Positive		Negative	Positive	
Age of caregiver	≤35 years	119	23	1	0.703	54	89	0.006 *	52	91	0.650
	≥36 years	77	77	1		50	39		35	54	
Educational level	Low than national average	159	23	1	0.124	89	94	0.02 *	67	116	0.589
	High than national average	37	11	1		15	34		20	29	
Income level	Decile I	184	32	2	989	100	118	0.378	81	137	0.431
	Decile II	11	2	0		4	9		5	8	
	Decile III	1	0	0		0	1		1	0	
Use of home remedies	No	60	9	2	0.579	32	37	0.758	28	41	0.528
	Yes	136	25	3		72	91		59	104	
Knowledge	Low	-	-	-	-	90	106	0.149	72	124	0.184
	Medium	-	-	-		12	22		13	21	
	High	-	-	-		2	0		2	0	
Attitudes	Negative	90	12	2	0.149	-	-	-	-	-	-
	Positive	106	22	0		-	-		-	-	
Practices	Negative	72	13	2	0.184	50	37	0.003 *	-	-	-
	Positive	124	21	0		54	91		-	-	

* Statistically significant.

## Data Availability

Data are available upon reasonable request form the corresponding author.

## Supplementary Materials

The following supporting information can be downloaded at: https://www.mdpi.com/article/10.3390/healthcare12030294/s1, KAP Questionnaire.
