# Supplementary material for: Knowledge, Attitudes, and Practices of Parents in the Use of Antibiotics: A Case Study in a Mexican Indigenous Community
Source: Healthcare (Basel). 2024 Jan 24;12(3):294. doi: 10.3390/healthcare12030294 (PMC10855187; doi:10.3390/healthcare12030294)
Supplement: Supplementary file 1 [file healthcare-12-00294-s001.zip › healthcare-2782602-supplementary.pdf]

CUESTIONARIO DE CONOCIMIENTO, ACTITUDES Y PRÁCTICAS DEL USO DE ANTIBIÓTICOS EN ÁREAS RURALES.

Nombre del niño: ___________________________________

Fecha de Investigación: ______________________________

No ____

Características Demográficas

1	Sexo del tutor: ①Hombre ②Mujer	
2	Edad del tutor:	
3	Nivel de educación del tutor:<br>① Analfabeta ② Escuela Primaria ③ Escuela Secundaria ④Preparatoria/ Escuela Técnica ⑤Universidad o Superior	
4	¿Cuál fue el último año que aprobó en el nivel del punto anterior?<br>① ② ③ ④ ⑤ ⑥	
5	Parentesco con el niño (a) :<br>① Padre ② Madre ③ Abuelo ④ Abuela ⑤Otro	
6	¿Quién se encarga del niño de manera diaria?<br>① Padre ② Madre ③ Abuelo ④ Abuela ⑤Otro	
6.1	En caso de que el niño(a) se enferme de una gripa o fiebre, ¿Quién decide si llevarlo al médico o no?<br>① Padre ② Madre ③ Padre y Madre ④Abuelo ⑤ Abuela ⑥Otro	
6.2	¿Si su hijo se llegará a enfermar cuanta autoridad tiene usted en decidir sobre la salud del niño(a)?<br>Ninguna autoridad [0 | 1 | 2 | 3 | 4 | 5 | 6 | 7 | 8 | 9 | 10] Toda la autoridad	

7	¿Cuáles son los ingresos mensuales de su familia?	
7.1	¿Cuánto dinero le dan los padres del niño (a)?________Pesos (Los abuelos responden esta pregunta)	
8	¿Cuántos integrantes tienen en la familia?	
9	Número de niños de 3 a 8 años:	
9.1	¿Cuántas habitaciones para dormir tiene la casa?	
**Condiciones generales del niño (a) que participa en el estudio**		
10	Edad del niño (a):	
11	Sexo del niño (a): ① Hombre ② Mujer	
12	¿Cuántas veces se enfermó el niño (a) en los pasados 6 meses?	
13	¿Qué enfermedades tuvo?<br>① Enf. Respiratorias ② Enf. Gastrointestinales ③Enf. respiratorias y gastrointestinales ④ Otras	
14	¿Cuáles son las instituciones de salud que usted prefiere para el cuidado de su hijo (a)?<br>① Clínica del Pueblo ② Centro de Salud de la Ciudad ③ Hospital ④ Farmacia ⑤ Otro	
15	¿Qué opina del estado de salud del menor?<br>Extremadamente malo | 0 | 10 | 20 | 30 | 40 | 50 | 60 | 70 | 80 | 90 | 100 | Extremadamente bueno	

Sección A Conocimientos

16. Nombre del medicamento	¿Alguna vez ha escuchado el nombre de este medicamento? ① Si ② No	¿Cree que es un antibiótico? ① Si ② No ③ No se	Nombre del medicamento	¿Alguna vez ha escuchado el nombre de este medicamento? ① Si ② No	¿Cree que es un antibiótico? ① Si ② No ③ No se	Nombre del medicamento	¿Alguna vez ha escuchado el nombre de este medicamento? ① Si ② No	¿Cree que es un antibiótico? ① Si ② No ③ No se
Ceftriaxona			Amoxicilina			Penicilina		
Cefradina			Azitromicina			Amikacina		
Cefalexina			Lincomicina			Metronidazol		
Cefuroxima			Eritromicina			Tinidazol		
Cefixina			Roxitromicina			Levofloxacino		

17	¿Está usted consiente que debe seguir las instrucciones que le dé el doctor cuando toma antibióticos?<br>①Totalmente en desacuerdo ② En desacuerdo ③ Indeciso ④De acuerdo ⑤Totalmente de acuerdo	
18	¿Sabía usted que el uso inapropiado de los antibióticos puede crear resistencia a los antibióticos?<br>①Totalmente en desacuerdo ② En desacuerdo ③ Indeciso ④De acuerdo ⑤Totalmente de acuerdo	
19	¿Sabía usted que es necesaria una receta para obtener antibióticos?<br>①Totalmente en desacuerdo ② En desacuerdo ③ Indeciso ④De acuerdo ⑤Totalmente de acuerdo	
20	¿Cuál es el principal medio de información sobre el uso de antibióticos? **(pueden seleccionar varias respuestas)**<br>① Doctores ② Seminarios ③ Farmacias ④ Televisión ⑤ Internet ⑥ Libros o revistas ⑦ Folleto del antibiótico ⑧ Otro	

Sección B Actitudes

21	¿Considera que el uso de los antibióticos puede evitar algunas enfermedades (como una gripa)?<br>①Totalmente en desacuerdo ② En desacuerdo ③ Indeciso ④De acuerdo ⑤Totalmente de acuerdo	
22	¿Considera que el uso de los antibióticos es demasiado en esta comunidad?<br>①Totalmente en desacuerdo ② En desacuerdo ③ Indeciso ④De acuerdo ⑤Totalmente de acuerdo	
23	¿Considera que es peligroso para los niños que se contagien con bacteria resistente a los antibióticos?<br>①Totalmente en desacuerdo ② En desacuerdo ③ Indeciso ④De acuerdo ⑤Totalmente de acuerdo	
24	¿Considera que el uso combinado de antibióticos tienen mejor efecto?<br>①Totalmente en desacuerdo ② En desacuerdo ③ Indeciso ④De acuerdo ⑤Totalmente de acuerdo	
25	¿Puede una persona parar de tomar el tratamiento completo de antibióticos si los síntomas han mejorado?<br>①Totalmente en desacuerdo ② En desacuerdo ③ Indeciso ④De acuerdo ⑤Totalmente de acuerdo	
26	¿Considera que los antibióticos más caros son mejores?<br>①Totalmente en desacuerdo ② En desacuerdo ③ Indeciso ④De acuerdo ⑤Totalmente de acuerdo	
27	¿Tomaría la iniciativa de pedir al médico que le recete el uso de antibióticos?<br>①Totalmente en desacuerdo ② En desacuerdo ③ Indeciso ④De acuerdo ⑤Totalmente de acuerdo	
28	¿Cree usted que debería ser necesario el uso de recetas médicas para poder comprar antibióticos?<br>①No necesario ② Poco necesario ③ Moderadamente necesario ⑤Muy necesario	

Considera necesario el uso de antibióticos si su hijo tiene una de las siguientes enfermedades:

29	Bronquitis	①Siempre ②Usualmente ③Algunas veces ④Nunca ⑤No se	
30	Diarrea	①Siempre ②Usualmente ③Algunas veces ④Nunca ⑤No se	
31	Infección de oído	①Siempre ②Usualmente ③Algunas veces ④Nunca ⑤No se	
32	Fiebre	①Siempre ②Usualmente ③Algunas veces ④Nunca ⑤No se	
33	Tos seca	①Siempre ②Usualmente ③Algunas veces ④Nunca ⑤No se	
34	Ardor de garganta	①Siempre ②Usualmente ③Algunas veces ④Nunca ⑤No se	
35	Nariz tapada	①Siempre ②Usualmente ③Algunas veces ④Nunca ⑤No se	

Sección C Practicas

36	¿Guarda antibióticos en su casa para sus hijos?<br>①Si ②No ③No estoy seguro si son antibióticos	
37	Por favor díganos el nombre del último antibiótico que uso.	
38	¿Dónde obtiene generalmente los antibióticos? **(pueden seleccionar varias respuestas)**<br>①Clínica del pueblo ②Centro de salud de la ciudad ③Hospital de la Ciudad ④Farmacia privada ⑤ Otro	
39	¿Con que frecuencia guarda los antibióticos cuando se enferma el niño (a)?<br>① Siempre ② Usualmente ③ Algunas veces ④ Raramente ⑤ Nunca	
40	¿Con que frecuencia usa antibióticos como prevención para evitar enfermedades como una gripa?<br>① Siempre ② Usualmente ③ Algunas veces ④ Raramente ⑤ Nunca	
41	Con que frecuencia sigue las instrucciones de el medico cuando le dan antibióticos al niño (a)<br>① Siempre ② Usualmente ③ Algunas veces ④ Raramente ⑤ Nunca	
42	¿Alguna vez ha aumentado la dosis del antibiótico para que sea más eficaz?<br>① Siempre ② Usualmente ③ Algunas veces ④ Raramente ⑤ Nunca	
43	¿Alguna vez ha reducido la dosis del antibiótico para evitar problemas futuros?<br>① Siempre ② Usualmente ③ Algunas veces ④ Raramente ⑤ Nunca	
44	¿Alguna vez ha usado más del antibiótico en su hijo (a)?<br>①Siempre ②Usualmente ③Algunas veces ④Raramente ⑤Nunca	
45	¿Alguna vez ha usado antibióticos de manera intermitente o suspendido y retomado el tratamiento para el niño (a)?<br>①Siempre ②Usualmente ③Algunas veces ④Raramente ⑤Nunca	
46	¿Alguna vez ha suspendido el uso de antibióticos cuando los síntomas han mejorado?<br>①Siempre ②Usualmente ③Algunas veces ④Raramente ⑤Nunca	
47	¿Alguna vez ha suspendido el tratamiento de antibióticos cuando los síntomas han desaparecido de su niño (a)?<br>①Siempre ②Usualmente ③Algunas veces ④Raramente ⑤Nunca	
48	¿Alguna vez ha continuado el tratamiento del uso de antibióticos, aunque los síntomas hayan desaparecido?<br>①Siempre ②Usualmente ③Algunas veces ④Raramente ⑤Nunca	
49	¿Qué factores considera cuando decide usar un antibiótico?<br>①Precio ②Marca ③La recomendación Médica ④La recomendación de otras personas	
50	¿Qué tipo de remedio casero utiliza para tratar la gripa?	
